# Integrated multidisciplinary approach to aneurysm hemodynamic analysis: numerical simulation, *in Vitro* experiment, and deep learning

**DOI:** 10.3389/fbioe.2025.1602190

**Published:** 2025-06-03

**Authors:** Tingting Fan, Jinhang Wang, Xu Wang, Xi Chen, Dongliang Zhao, Fengjie Xie, Guangxin Chen

**Affiliations:** ^1^ School of Biomedical Engineering, Capital Medical University, Beijing, China; ^2^ Beijing Institute of Heart, Lung, and Blood Vessel Diseases, Beijing, China; ^3^ Emergency and Critical Care Center, Beijing Anzhen Hospital, Capital Medical University, Beijing, China; ^4^ Peking University Shenzhen Graduate School, Shenzhen Bay Laboratory, Shenzhen, Guangdong, China; ^5^ Department of Critical Care Medicine, Hongqi Hospital Affiliated to Mudanjiang Medical University, Mudanjiang, Heilongjiang, China; ^6^ Medical Image College, Mudanjiang Medical University, Mudanjiang, Heilongjiang, China

**Keywords:** aneurysm, hemodynamic, numerical simulation, deep learning, in vitro experiment

## Abstract

Aneurysm, as life-threatening vascular pathologies, are significantly influenced by hemodynamic factors in their development. The combine of numerical simulation and *in vitro* experiment have laid the foundation for high-precision hemodynamic analysis, while the integration of deep learning technologies has significantly enhanced computational efficiency. However, current researches still face challenges such as limitations in biomimetic materials, and incomplete understanding of mechano-biological coupling mechanisms. In this review, we systematize traditional and emerging methodologies characterizing hemodynamic perturbations across the pathophysiological continuum of aneurysmal expansion, rupture, and thrombosis progression. This review aims to (1) elucidate mechanistic underpinnings of aneurysm destabilization, (2) inspire people to establish standardized quantification protocols for hemodynamic analysis, and (3) pave the way for patient-specific risk stratification enabling data-driven clinical interventions.

## 1 Introduction

Aneurysms, as a potentially fatal vascular disease, have always been a focus of research in the field of cardiovascular and cerebrovascular diseases. An aneurysm is defined as a pathological bulging that occurs locally in the artery wall. It encompasses multiple types and is typically classified according to anatomical location. These include IAs (Ferns et al., 2011), carotid artery aneurysms (Feng et al., 2020; Welleweerd et al., 2015), CAA (Angelini, 2007), TAA (Senser et al., 2021), AAA (Anagnostakos and Lal, 2021), popliteal artery aneurysms (Jergovic et al., 2022), and other less common site-specific expansive lesions.

Although aneurysms occurring in various anatomical sites all involve structural changes in the arterial wall, they differ significantly in terms of incidence, pathogenesis, and prognosis (Camasão and Mantovani, 2021; Yang et al., 2020). IAs have an incidence rate of approximately 3%–5% in the general population. Such aneurysms typically arise due to abnormal WSS or genetic factors, such as mutations, leading to endothelial cell damage, disruption of the elastic membrane, and subsequent local inflammatory reactions, ultimately weakening the arterial wall (Chalouhi et al., 2013; Ferns et al., 2011; Miyata et al., 2022; Leemans et al., 2019; Sekhar and Heros, 1981). The most severe consequence of IAs is rupture, resulting in subarachnoid hemorrhage and poor clinical outcomes (CHMAYSSANI et al., 2011; Hadad et al., 2024; Hejazi and Phani, 2022). CAA are relatively rare, with an incidence rate between 0.3% and 4.9% among patients undergoing coronary angiography. Atherosclerosis is the most common cause of CAA, involving damage to the medial layer of the vessel wall and breakdown of elastic fibers (Serruys et al., 2023; Gutierrez et al., 2017; Iemura et al., 2000; Gellis et al., 2022). Such aneurysms may lead to severe cardiovascular events, including myocardial infarction and arrhythmia (Taskesen et al., 2021; Conrad et al., 2024). TAA have an annual incidence rate of approximately six per 100,000 individuals and are closely associated with genetic conditions such as Marfan syndrome and Loeys-Dietz syndrome (Senser et al., 2021). These genetic disorders accelerate degenerative changes in the aortic wall by affecting the stability of collagen and elastin proteins. TAA often remain undetected until symptomatic, and rupture results in high mortality. AAA are more common in elderly males over 65 years, with approximately 8% prevalence in this population (Hellmann et al., 2007; Bossone and Eagle, 2021; Heussel et al., 1997). Smoking, hypertension, and atherosclerosis are major risk factors (Tang et al., 2005; Wang et al., 2023). The mortality rate following AAA rupture is exceedingly high, making rupture prevention a primary focus in AAA management (Wang et al., 2024). The risk of rupture increases with aneurysm diameter, and surgical intervention is recommended particularly when the diameter exceeds 5.5 cm (Anagnostakos and Lal, 2021). Despite the differences in clinical manifestations and risk factors, aneurysms across various anatomical sites pose significant threats to patient health and survival. Understanding these diseases contributes to improving diagnostic accuracy and treatment efficiency, reducing patient mortality risks.

Aneurysms occurring at different anatomical locations, differ markedly not only in clinical manifestations but also in lesion structure, flow patterns, and surrounding tissue environments, presenting substantial challenges for precise diagnosis and individualized treatment. Throughout aneurysm formation, expansion, and potential rupture, mechanical factors play indispensable roles. The development of aneurysms is closely related to mechanical factors such as WSS, circumferential stress, and flow stagnation. These factors accelerate aneurysm expansion and rupture by damaging vascular endothelial cells, promoting collagen degradation, or increasing thrombosis risk. Abnormal variations in WSS can cause endothelial cell injury, increased circumferential stress may lead to collagen degradation and vessel wall remodeling, while flow stagnation and vortices frequently induce thrombosis and embolization (Frösen et al., 2019). In CAA, abnormal WSS can weaken the arterial wall, causing localized vessel dilation and elevating rupture risk. In IAs, studies indicate that abnormal hemodynamics, particularly elevated shear stress, are strongly associated with aneurysm formation and rupture. Areas of flow stagnation in IAs, especially at arterial bifurcations, are prone to thrombosis, and subsequent embolization can result in severe complications. Meanwhile, AAA typically involve expansion and weakening of the abdominal aortic wall, closely associated with local changes in circumferential and shear stress. In AAA, elevated circumferential stress can exceed the mechanical strength of the arterial wall, potentially leading to aneurysm rupture.

**FIGURE 1:**
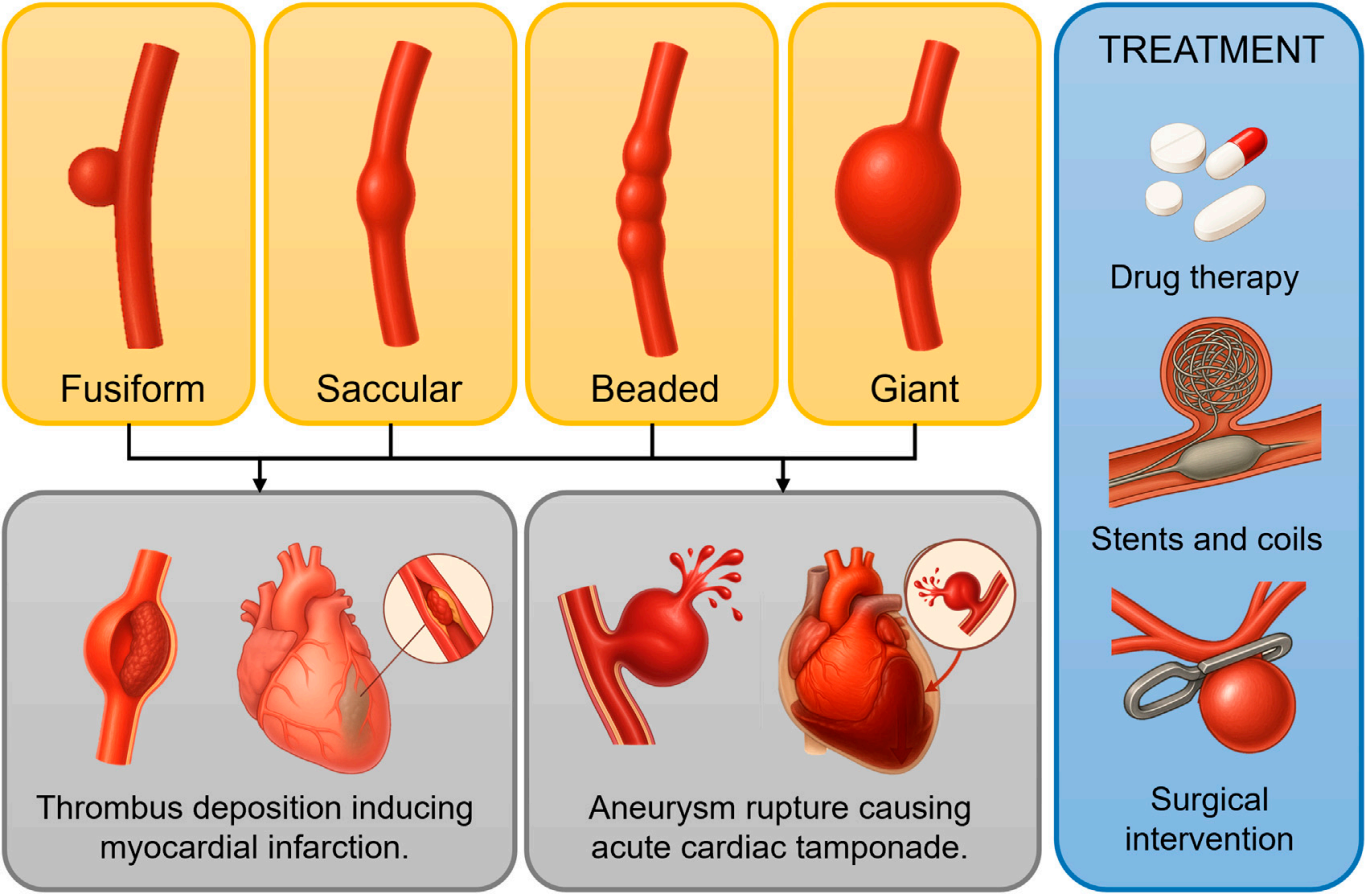
Morphological classification of coronary artery aneurysms (CAAs) and adverse events associated with aneurysm progression. The yellow box shows four morphological classifications of CAAs; the gray box illustrates potential cardiovascular adverse events resulting from thrombus deposition, growth, and rupture; the blue box presents commonly used treatment strategies.

In summary, current mechanical studies on aneurysmal disease primarily focus on aneurysm expansion, rupture, and thrombosis deposition. Alterations in the hemodynamic environment significantly influence aneurysm expansion rate, rupture risk, and thrombosis formation, dictating disease progression. Thus, elucidating the mechanical mechanisms underlying aneurysm pathogenesis and progression to guide precise intervention strategies remains an urgent issue in cardiovascular research. Figure 1 illustrates the morphological classification of CAA, along with related adverse cardiovascular events and common treatment options. This classification is broadly similar to that of IAs and AAA, which also include dissecting and pseudoaneurysm types. Treatment options for AAA include EVAR, open surgery, and medication to control blood pressure and limit aneurysm growth.

Therefore, this review will focus on recent advances and challenges in aneurysm-related hemodynamic research, including numerical simulations, *in vitro* experimental measurements, and artificial intelligence-based mechanical analysis methods. Additionally, we will compare the mechanical heterogeneity among aneurysms at different anatomical locations, explore the potential and limitations of various research methods in clinical translation, and propose critical future research directions aimed at enhancing aneurysm diagnosis and treatment.

## 2 Mechanistic insights into aneurysm progression

### 2.1 Morphology and mechanics in aneurysm expansion

The coupling between morphological features of aneurysms and local hemodynamics is a critical driver for aneurysm initiation, progression, and rupture. According to anatomical location, aneurysms are classified into CAA, IAs, and AAA. Based on morphological features, they are categorized into fusiform and saccular aneurysms. Common geometric parameters in current studies include directly measured maximum aneurysm diameter (*D*_max_), length (*L*), volume (*V*), surface area (*A*), curvature (*τ*), torsion (*κ*), neck width, neck diameter (*D*_*Neck*_), neck angle, and tilt angle (*θ*). Three-dimensional vascular geometries are acquired through various imaging techniques such as CTA, MRA, and DSA. To enhance measurement accuracy, two-dimensional image measurement via multiplanar reconstruction from CT images is often cross-validated with three-dimensional geometry measurements. In addition to directly measured geometric parameters, researchers calculate ratio-based parameters like ASI, SR, aspect ratio, sphericity index, UI, bifurcation angle, CE, and LE. Generally, aneurysm morphologies vary across locations, and specific geometric features are more suitable for different aneurysm types. Researchers must select relevant parameters according to their specific research context.

**FIGURE 2:**
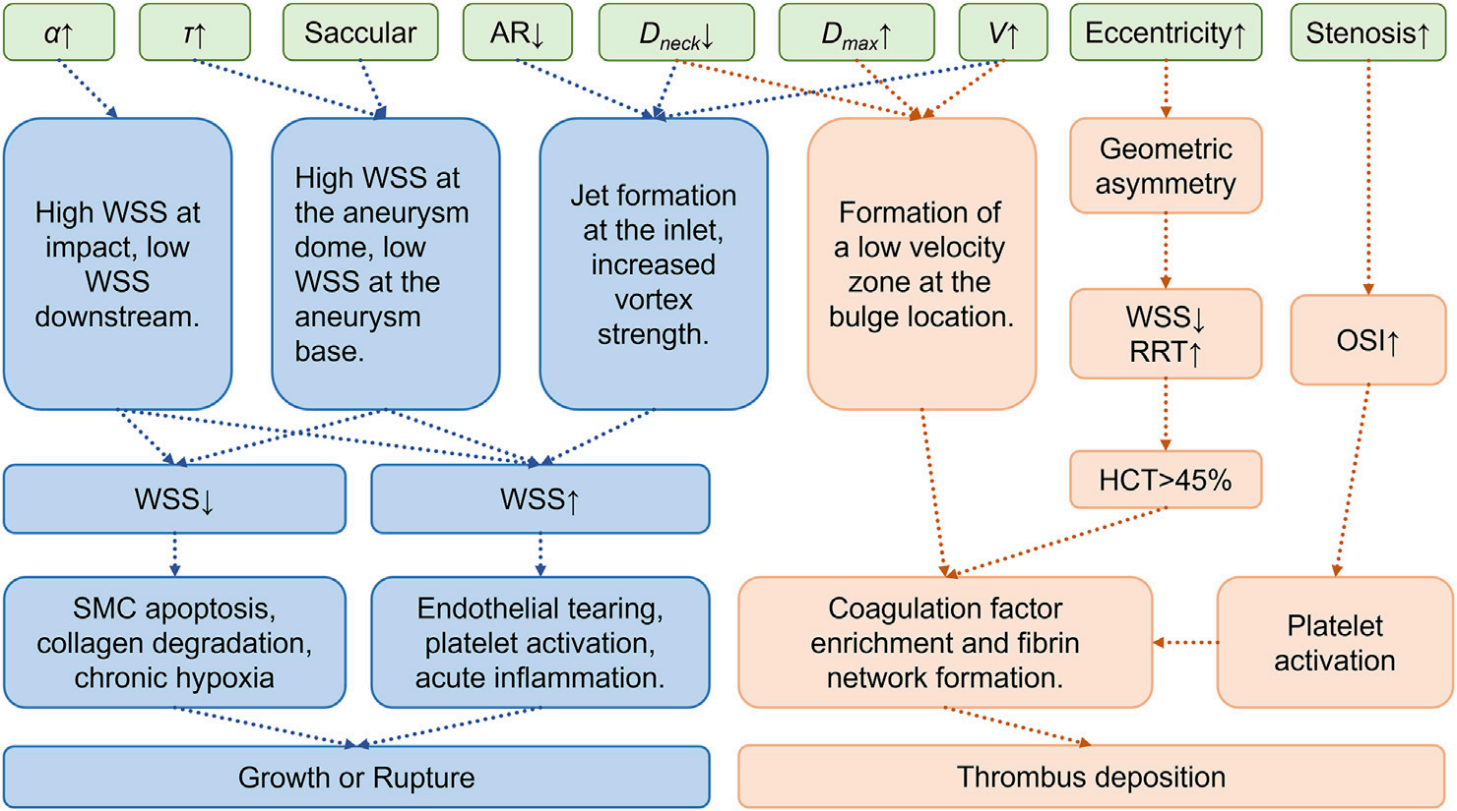
Morphological-mechanical-biological coupling mechanism of aneurysm growth, rupture, and thrombus deposition.Green indicates geometric parameters, blue indicates mechanisms of growth and rupture, and orange indicates mechanisms of thrombus deposition.*α*: Bifurcation angle, *τ*: Curvature, AR: Aspect Ratio, *D*_max_: Maximum aneurysm diameter, *D*_*neck*_: Neck diameter, *V*: Aneurysm volume, HCT: Hematocrit.

Significant coupling relationships exist between aneurysm geometric parameters and local hemodynamics, and these couplings strongly influence aneurysm expansion. Taking CAA an example, larger aneurysm diameters and volumes typically correlate with reduced TAWSS, which has been linked to accelerated luminal dilatation. Increased aneurysm size also corresponds to higher OSI and RRT, thus aggravating disturbed flow that facilitates wall remodeling and continued enlargement (Zhang et al., 2024). Fusiform aneurysms, characterized by axial elongation, generally experience less flow disturbance yet may still undergo progressive enlargement under sustained circumferential wall stress. In contrast, saccular aneurysms exhibit pronounced flow separation and vortical flow fields due to their distinct neck structures, a pattern associated with localized wall weakening and outward bulging (Rafiei and Saidi, 2022; Zhang et al., 2024). For instance, studies on IAs show substantial reductions in intra-aneurysmal velocity and vortex formation, causing significant enlargement of low-WSS regions that precede measurable diameter growth (Hoi et al., 2004; Hadad et al., 2024). Similarly, in CAA, substantial expansion or neck constriction commonly leads to low-velocity flow areas and widespread low-WSS distribution, further accelerating mural remodeling and progressive dilatation (Singhal and Gupta, 2024; Wong et al., 2020; Hoi et al., 2004). Long-term follow-up of AAA cohorts has revealed that patients exposed to persistently low TAWSS (<0.4 Pa) exhibit mean growth rates exceeding 0.8 mm year^-1^, underscoring the clinical relevance of the hemodynamic–morphology coupling described above (Bappoo et al., 2021; Faisal et al., 2025). Table 1 summarizes current research on the relationships between morphological parameters and hemodynamic indicators. Figure 2 summarizes the current research progress on the geometryss–mechanicss–biology interplay in aneurysms, illustrating how different geometric features influence the development of aneurysmal disease.

**TABLE 1:** Research progress on the relationship between morphological parameters and hemodynamic indicators.

Location	Model	Extraction method	Morphological parameters	Impact on disease	References
CAA	Real model	CTA	Saccular or fusiform	Saccular aneurysms are more prone to rupture, while fusiform aneurysms are more likely to accumulate thrombi	Zhang et al. (2024)
IAs	Real model	DSA, CTA, MRA	AIRC	The more pronounced the AIRC, the more likely IAs are to grow and rupture	Miyata et al. (2022)
IAs	Real model	3DRA	SR, VOR, NSI, CR	The rupture of IAs can be predicted jointly by morphological indices, flow, and WSS	Hadad et al. (2024)
AAA,TAA	Ideal model	-	Degree of kinking	Kinking may lead to the growth of an aneurysm	Hejazi and Phani (2022)
CAA	Real model	CTA	*V*/*V*_*0*_	Large size leads to thrombus deposition in aneurysms	Rafiei and Saidi (2022)
AAA	Real model	CTA	*D*_max_, growth rate	The faster the growth rate, the more likely it is to lead to rupture	Anagnostakos and Lal (2021)
TAA	Real model	CTA	*D*_max_, growth rate	The faster the growth rate, the more likely it is to lead to rupture	Senser et al. (2021)
IAs	Real model	CTA	Relative volume change (RVC)	Changes in RVC can show the range of motion of IAs, thereby assessing the risk of rupture	Stam et al. (2021)
CAA	Ideal model and Real model	CTA	*τ*(Curvature), Degree of stenosis	The degree of curvature and narrowing can promote plaque formation	Wong et al. (2020)
AAA	Real model	CTA	Stagnation Zone Volume	An increase in the stagnation zone is associated with the growth and rupture of AAA	Joly et al. (2018)
IAs	Real model	3D DSA	DNR (Dome-Neck Ratio)	The size of the aneurysm dome is negatively correlated with the blood flow velocity and WSS within the IAs	Tateshima et al. (2010)
IAs	Ideal model	-	daughter aneurysms	The development of daughter aneurysms has a temporary protective effect	Meng et al. (2013)
IAs	Ideal model	-	*τ*	Lateral saccular aneurysms located on more curved arteries are subjected to higher stress	Hoi et al. (2004)

Currently, CFD numerical simulations are the primary research method, quantitatively analyzing aneurysm hemodynamics through solving velocity, pressure, and WSS-related fields. Additional indices, such as helicity and cross-flow index (CFI), evaluate flow multidirectionality and turbulence’s effects on aneurysm walls, enriching morphology-dynamics coupling analyses. As research advances, multi-scale patient-specific modeling and precise CFD simulations become prevalent, enhancing morphological parameters’ value for clinical risk prediction and therapeutic strategy development.

### 2.2 Hemodynamic of aneurysm rupture

Aneurysm rupture is a complex process involving multiple mechanical and biological mechanisms. Endothelial dysfunction and abnormal local blood flow—characterized by pronounced flow deceleration, recirculation, and vortices—create hemodynamic environments that lower WSS and raise OSI, ILT formation and setting the stage for wall failure (Wang et al., 2024; Al-Jumaily et al., 2023). These abnormal flow patterns decrease WSS and increase OSI, promoting thrombus formation and reducing endothelial cell viability. Low-WSS areas undergo chronic hypoxia, triggering inflammatory cell aggregation, elevated MMP expression, and apoptosis, ultimately causing arterial wall structural degeneration, including elastin and collagen degradation, thereby critically weakening the wall and predisposing it to rupture (Arslan and Salman, 2023; Belkacemi et al., 2023; Philip et al., 2022). Enlarged aneurysm size is often accompanied by markedly reduced TAWSS and elevated OSI and RRT; these hemodynamic changes promote ILT deposition, intensify wall hypoxia, and therefore markedly elevate rupture risk, as demonstrated for CAA, IAs, and AAA (Zhang et al., 2024). In AAA, rupture usually occurs in weakened wall regions such as posterior or lateral walls (Singh et al., 2021; Golledge, 2019), whereas IAs commonly rupture at inflow impingement sites or arterial bifurcations (Hadad et al., 2024; Miyata et al., 2022). Saccular morphologies with narrow necks concentrate inflow jets, generating high-WSS impingement zones that can coexist with surrounding low-WSS regions, collectively accelerating structural degradation and precipitating rupture (Rafiei and Saidi, 2022). Conversely, fusiform aneurysms may rupture after prolonged periods of progressive dilatation when mural stress exceeds tensile strength despite relatively smoother core flow patterns. Peak wall stress (PWS) plays a critical role in rupture events, with high-PWS areas indicating mechanical stress concentration, increasing local degeneration and rupture likelihood (Singh et al., 2021).

The role of shear stress in aneurysm rupture remains controversial. The high WSS hypothesis suggests that high shear forces, particularly in IAs, directly damage endothelial cells, triggering acute inflammatory responses and increasing rupture risk (Zhang et al., 2019; Wang et al., 2018; Cho, 2023). Conversely, the low WSS hypothesis emphasizes chronic pathologies induced by low shear regions, particularly in AAA, where long-term hypoxia-driven matrix degradation and endothelial dysfunction are primary rupture triggers (Zhang et al., 2016; Miura et al., 2013; Boyd et al., 2016; Zhou et al., 2017). Recent studies indicate significantly increased rupture risks when WSS exceeds a threshold (12.3 dyne/cm^2^), especially in anterior communicating artery aneurysms (ACoA), with rupture risks multiplying per unit increase of WSS (Miura et al., 2013). High WSS regions involve concentrated flow impingement, mechanical endothelial damage, and significant inflammatory responses, accelerating MMP release and matrix degradation, ultimately compromising wall strength and structural integrity. Low WSS mechanisms first manifest as reduced and oscillatory blood flow (increased OSI), promoting ILT formation (Wang et al., 2018). Thrombus deposition exacerbates wall hypoxia, endothelial dysfunction, and chronic inflammation, including macrophage and neutrophil aggregation and MMP release, accelerating vascular matrix degradation and smooth muscle cell apoptosis (Zhang et al., 2019). Moreover, low WSS diminishes endothelial protective functions by reducing NO production, exacerbating inflammation and vessel wall fragility (Miura et al., 2013; Zhang et al., 2016). Numerical simulations and clinical evidence further suggest chronic low WSS environments induce inflammation and hypoxia, structurally damaging aneurysm walls, ultimately causing mechanical instability and rupture under repetitive blood pressure fluctuations (Boyd et al., 2016; Zhou et al., 2017). However, studies also highlight dynamic changes in relationships between WSS magnitude and aneurysm expansion and rupture. Early aneurysm expansion stages might exhibit high WSS, transitioning to low WSS dominance as geometry evolves, forming chronic hypoxia and inflammatory conditions, ultimately leading to rupture (Singh et al., 2021; Wang et al., 2018; Zhou et al., 2017; Cho, 2023). This shift from high to low WSS underscores WSS’s varying influence across aneurysm development stages.

Although CFD and FSI numerical simulations have significantly advanced our understanding of aneurysmal hemodynamics, how precisely high and low WSS differentially drive aneurysm expansion and rupture remains unclear, necessitating integrated research involving clinical imaging and pathology for further elucidation.

### 2.3 Thrombus deposition dynamics

The formation of ILT is a pathological process influenced by complex hemodynamic and biological mechanisms (Stark and Massberg, 2021; Hathcock, 2006; Mihalko and Brown, 2020). Currently, two distinct mechanical hypotheses, namely, the low-WSS hypothesis and the high-WSS hypothesis, explain aneurysm thrombosis formation, each with significant mechanistic differences and ongoing debates.

The low-WSS hypothesis emphasizes that regions of significantly reduced or stagnant blood flow within aneurysms often accompany vortex, recirculation, and swirl flow structures (Buck et al., 2018; Deplano and Siouffi, 1999). For example, Cao et al. performed numerical simulations on Kawasaki Disease (KD) coronary artery aneurysms, demonstrating that low TAWSS, high OSI, and high RRT significantly increased thrombotic risk, with thrombi frequently located at the proximal and myocardial sides of aneurysms (Cao et al., 2023). Additionally, another study by Cao et al. (2022) indicated that the combined presence of low TAWSS, high OSI, and high RRT notably enhanced thrombus formation risks in KD coronary aneurysms. Under low-WSS conditions, the blood velocity is extremely low, extending the residence time of RBC, platelets, and coagulation factors (Van der Waerden et al., 2025; Millon et al., 2015). Prolonged stagnation decreases local oxygen levels, triggering endothelial cell dysfunction characterized by significantly diminished secretion of antithrombotic substances like NO and prostacyclin (PGI2), thereby reducing normal anticoagulant function. Hypoxia and prolonged low shear stress enhance endothelial cell expression of adhesion molecules, intensifying platelet-endothelial adhesion (Millon et al., 2015). Stagnant blood flow results in the local accumulation of coagulation factors, triggering and amplifying the coagulation cascade reaction, eventually forming stable fibrin networks and progressively larger thrombotic structures (Zhou et al., 2023).

The high-WSS hypothesis emphasizes abnormally high shear forces at aneurysm entrances or local stenotic areas, leading to endothelial cell damage and rapid platelet adhesion and aggregation (Casa and Ku, 2017). According to the high-WSS theory, aneurysm entrances or localized narrow regions exhibit significantly elevated blood flow velocity and shear stress (Dolan et al., 2013). High shear conditions continuously expose endothelial cells to mechanical stimuli, resulting in mechanical damage or activation (Sho et al., 2002; Masuda et al., 1999; Sho et al., 2003). Under high shear stress, vWF molecules undergo mechanical stretching, increasing binding sites for platelet glycoprotein receptors (GP Ib), significantly enhancing rapid platelet adhesion and aggregation onto damaged endothelium, forming initial platelet-rich thrombotic cores (Tada et al., 2011; Tada et al., 2010). Furthermore, endothelial injury exposes procoagulant substances such as subendothelial collagen fibers, rapidly initiating thrombogenesis. Sustained mechanical stress and endothelial damage trigger local inflammatory responses, releasing inflammatory mediators (such as ADP) and tissue factors, further accelerating the coagulation cascade.

The low-WSS and high-WSS mechanisms differ in focus: low-WSS emphasizes gradual thrombus deposition due to blood stagnation, whereas high-WSS highlights rapid initial platelet aggregation due to mechanical injury. Although both mechanisms likely coexist clinically, detailed exploration of their respective mechanisms and clinical significance is crucial for comprehensively understanding aneurysm thrombosis and developing precise intervention strategies.

The mechanism of thrombus deposition also demonstrates significant anatomical specificity. For example, in intracranial aneurysms, dynamic thrombus deposition and shedding directly influence downstream embolization risk (Ridker and Rane, 2021; Malek et al., 1999; Deplano and Siouffi, 1999). Conversely, thrombus deposition in AAA temporarily alleviates mechanical stress on aneurysm walls but may eventually contribute to further wall weakening (Wang et al., 2023; Xu et al., 2023; Hayashi et al., 2006). Studies on abnormal flow parameters and thrombus formation in coronary artery aneurysms have gained considerable attention. For instance, Woźniak et al. (2024) confirmed significant associations between low WSS, high OSI, high RRT, and thrombus deposition in coronary aneurysms.

### 2.4 Risk stratification of aneurysms

Aneurysm risk stratification in contemporary clinical practice has evolved from a “one-size-fits-all” diameter paradigm toward nuanced, disease-specific schemes that integrate demographics, imaging surrogates and, increasingly, biologic read-outs of wall vulnerability.

For IAs, prospective outcome cohorts underpin three complementary tools. The PHASES score translates six readily available variables—population, hypertension, age, diameter, prior subarachnoid haemorrhage and site—into an absolute 5-year rupture probability that ranges from <0.5% (score 0–2) to ≈18% (score ≥13), and is now embedded in both European and North-American guidelines (Greving et al., 2014). Growth prediction is addressed by the ELAPSS score, in which history of rupture, aneurysm location, age, population, morphology and size yield a 5-year enlargement risk exceeding 10% once the sum reaches 12 points; this facilitates personalized surveillance intervals and early endovascular referral (Backes et al., 2017). When therapeutic equipoise persists, the multidisciplinary UIATS consensus model quantifies 29 patient-, aneurysm- and treatment-related factors on two opposing columns; a net difference of ±3 provides a reproducible threshold either for intervention or watchful waiting (Etminan et al., 2015; Backes et al., 2017). Beyond morphology, high-resolution vessel-wall MRI has introduced the aneurysm-to-pituitary-stalk contrast ratio; a value ≥0.5 denotes circumferential wall enhancement that correlates with inflammatory cell infiltration and identifies unstable lesions even when diameter is small (Wu et al., 2022).

For AAA, the maximal anteroposterior diameter remains the cornerstone: elective repair is advocated at ≥55 mm in men and ≥50 mm in women, or earlier when yearly growth surpasses 10 mm, as codified by the 2022 Society for Vascular Surgery guideline update (Chaikof et al., 2018). Yet diameter alone incompletely captures wall frailty. Finite-element modelling shows that a PWS >200 kPa—or, more sensitively, a peak wall rupture index (PWRI) elevated relative to patient-specific wall strength—distinguishes ruptured from size-matched intact aneurysms (Singh et al., 2021)​. Concomitantly, the burden and geometry of intraluminal thrombus have emerged as pivotal modifiers: posterior thrombus thickness >10 mm or a volumetric occupancy >40% accelerates hypoxic medial degeneration and triples rupture odds despite lowering computed wall stress, underscoring the dual biomechanical-biological nature of this substrate (Haller et al., 2018). Thoracic aortic disease follows a size-indexed logic tempered by genotype and growth rate. In patients with tricuspid aortic valves the current ACC/AHA statement recommends surgery once the ascending aorta reaches 55 mm, but lowers the threshold to 50 mm—or an aortic cross-sectional area/height ratio >10 cm^2^ m^-1^—for Marfan, Loeys-Dietz or familial forms, and mandates expedited repair when expansion exceeds 3 mm year^-1^ (Isselbacher et al., 2022).

Risk models for CAA secondary to KD rely on body-surface-normalised Z-scores: aneurysms with Z < 5 usually regress; those with Z 5–10 persist and entail chronic antiplatelet therapy; and “giant” lesions (Z ≥ 10 or absolute diameter ≥8 mm) carry a ≥20% 5-year thrombosis/myocardial-infarction risk, warranting lifelong anticoagulation and advanced imaging surveillance (Kato et al., 2023; McCrindle et al., 2017).

Collectively, these disease-specific stratification frameworks illustrate how clinical decision-making now weaves together absolute size, dynamic growth, composite risk scores, biomechanical metrics and imaging biomarkers to delineate a personalised trajectory from benign dilatation to catastrophic rupture or occlusion.

## 3 Current status of research methods on hemodynamic characteristics

### 3.1 Numerical simulations

**FIGURE 3:**
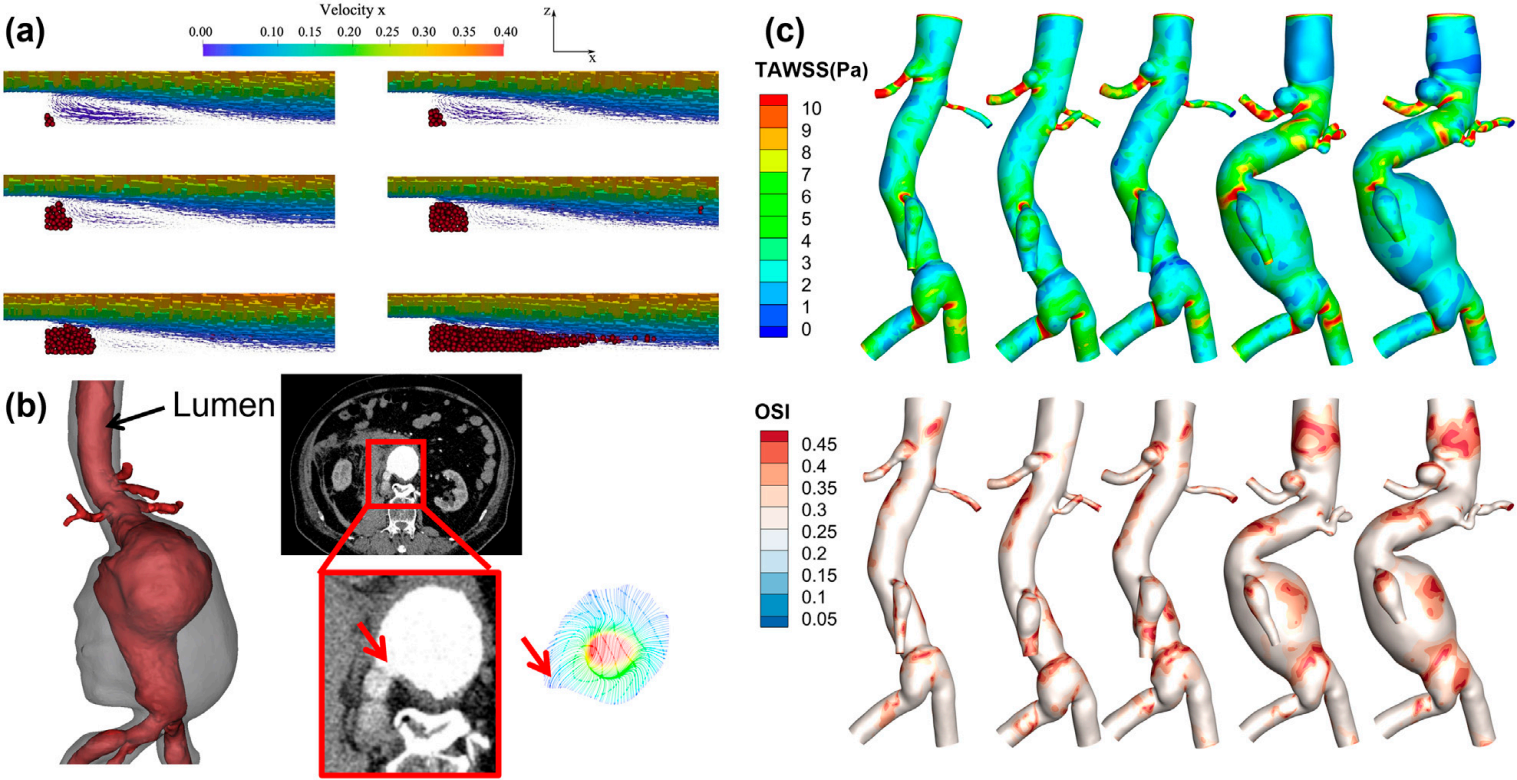
Numerical simulation-based study of thrombus formation and the progression and rupture of aneurysms. **(a)** is based on the Smoothed Particle Hydrodynamics method for simulating thrombus deposition processes in a backward step model (Monteleone et al., 2023); **(b)** Velocity field simulation at the rupture site of an abdominal aortic aneurysm, with the red arrow indicating the rupture point; **(c)** Aneurysm growth over five follow-ups in the same patient, along with contour plots of TAWSS and OSI.

CFD or FSI simulations are common tools to characterize hemodynamic properties within aneurysms. Recently, CFD methods have been widely applied in cardiovascular diseases such as coronary artery stenosis, aortic aneurysms, and cerebral aneurysms (Feng et al., 2020; Fan et al., 2019a; Feng et al., 2018). Figure 3 summarizes representative studies based on numerical simulation methods that investigate thrombus deposition, expansion, and rupture of aneurysms, including both idealized models and patient-specific vascular models.

The standard procedure for CFD simulations typically begins with the three-dimensional reconstruction of arterial geometry using medical imaging data (e.g., CTA, MRI, Micro-CT, or DSA) to obtain patient-specific anatomical models. The accuracy of vascular geometry reconstruction directly influences simulation outcomes. Multi-threshold segmentation techniques combined with manual corrections are generally employed to ensure geometric models are consistent with actual anatomical structures. During the meshing process, tetrahedral or polyhedral meshes are frequently used, supplemented by prism layer refinement to adequately capture wall shear layers and near-wall turbulent characteristics. The mesh element count typically ranges from hundreds of thousands to millions. However, current mesh sizes are often limited to scales of several hundred micrometers, which may fail to accurately capture small-scale flow features, particularly in regions sensitive to WSS, such as aneurysm cavities or vascular bifurcations, potentially introducing errors (Ren et al., 2022; Le et al., 2013; Kandangwa et al., 2022). In selecting algorithms for hemodynamic simulations, CFD usually employs the FVM to solve Navier–Stokes equations. Common spatial discretization schemes include the second-order upwind scheme and central difference method. Typical temporal integration methods include semi-implicit Euler schemes, BDF2, and other high-order implicit or semi-implicit methods to ensure simulation stability and accuracy. In complex flow regions such as AAA or intracranial aneurysms, LES methods are also utilized to capture richer turbulent structures (Vergara et al., 2017; Lancellotti et al., 2017).

Regarding the assumption of blood fluid properties, most studies still adopt the Newtonian fluid assumption, considering blood viscosity as a constant value (approximately 4–4.5 mPa·s) (Feng et al., 2020; Fan et al., 2019b). However, studies indicate that this simplification might introduce significant errors in low-flow regions or aneurysm vortex areas, failing to accurately simulate shear-thinning effects of blood, thereby affecting precise calculation of parameters like WSS and turbulence indices. Consequently, Non-Newtonian fluid models (such as Carreau or Carreau-Yasuda models) are gaining attention in research to more accurately represent blood rheological properties (Salman et al., 2019; Nørgaard et al., 2014; Miranda et al., 2021).

Currently, simulations commonly assume arterial walls as rigid structures, neglecting their actual deformable characteristics. This assumption is clearly limited, particularly given the significant histological differences between arterial regions. For example, coronary artery walls are relatively thin with notable tissue elasticity; cerebral artery walls, rich in elastic fibers, are relatively fragile; and aortic walls have a thicker elastic medial structure. Neglecting these tissue characteristics and structural differences can lead to errors in predicting hemodynamic parameters (e.g., pressure gradients, WSS, vortex stability). To enhance simulation accuracy, FSI methods have increasingly become a research trend. Unlike traditional CFD, which focuses solely on blood flow, FSI couples interactions between blood and arterial walls, thereby providing more realistic blood flow-wall deformation interaction models (Mendez et al., 2018; Lin et al., 2017). Typical FSI applications include stress redistribution after stent implantation, aortic valve opening and closing processes, and aneurysm wall rupture risk prediction (Pavlin-Premrl et al., 2021; Miranda et al., 2021; Pinho et al., 2019). For example, after coronary stent implantation, FSI can more accurately simulate stress distribution between the stent and vascular wall and the vessel remodeling process, offering more clinically valuable evaluations.

In determining boundary conditions, traditional methods commonly employ typical pressure/flow waveforms of the aorta or coronary arteries from clinical measurements or literature as inlet conditions (Huo et al., 2012; Cao et al., 2021; Tang et al., 2020; Salman et al., 2019). Outlet conditions generally adopt zero-pressure or simple linear resistance models. However, these simplified boundary conditions cannot fully reflect the physiological characteristics of the actual microcirculation or downstream vascular bed, possibly leading to over- or under-estimations of local hemodynamic characteristics. Some studies have begun incorporating more physiological Windkessel or other distributed parameter models to obtain more precise simulation results at outlet boundary conditions (Wang et al., 2023; Van der Horst et al., 2013; Sommer et al., 2022; Mei et al., 2020).

Concerning hemodynamic parameters, current studies mainly focus on WSS, TAWSS, OSI, RRT, vortex criteria (e.g., Q-criterion), ECAP, EL, among others. Accurate selection and prediction of these parameters have significant implications for clinical risk assessment. Table 2 summarizes commonly used hemodynamic indices, their formulas, and physiological significance. The accurate prediction of hemodynamic indices strongly depends on mesh precision, fluid model selection, wall boundary conditions, and outlet condition settings, and their reliability requires further validation. Present simulation method limitations mainly manifest in two areas: firstly, excessive dependence on boundary conditions, where uncertainties or simplifications in inlet and outlet conditions might skew results; secondly, insufficient clinical measurements to rigorously validate numerical simulation outcomes. Thus, future research should enhance coupling validation between experimental and numerical simulations, fully integrating medical imaging data, flow and pressure measurements, and physiological parameters to develop more refined FSI models and boundary conditions.

**TABLE 2:** Commonly used hemodynamic parameter formulas and their physiological significance.

Parameter	Calculation formula	Dimension	Physiological significance
TAWSS	$\text{TAWSS}=\frac{1}{T}\;\int_{0}^{T}\left|\tau_{w}\left(t\right)\right|\;dt$	Pa	Characterizes the average level of WSS over a cardiac cycle, reflecting the extent to which the vessel is subjected to long-term ‘squeezing’ effects. Both excessively low and high TAWSS are considered to be associated with the occurrence and development of diseases
OSI	$OSI=\frac{1}{2}\left(1-\frac{\left.\left|\int_{0}^{T}\tau_{w}\left(t\right)\;dt\right.\right|}{\int_{0}^{T}\left|\tau_{w}\left(t\right)\right|\;dt}\right)$	Dimensionless	Depicts the degree of oscillation of the wall shear stress direction within a cycle. Values closer to 0.5 indicate more frequent reversals of shear stress direction, often associated with areas susceptible to atherosclerosis or aneurysm instability
RRT	$RRT=\frac{1}{TAWSS\;\left(1-2\;OSI\right)}$	1/Pa	RRT characterizes regions where wall shear stress is both low and oscillates severely, providing a good marker for atherosclerotic lesions and areas prone to thrombosis
transWSS	$transWSS=\frac{1}{T}\int_{0}^{T}\left.\left|\tau_{w}\left(t\right)-\left(\tau_{w}\left(t\right)\cdot \hat{e_{m}}\right)\hat{e_{m}}\right.\right|\;dt$ $\hat{e_{m}}=\frac{\tau_{m}}{\left|\tau_{m}\right|},\tau_{m}=\frac{1}{T}\int_{0}^{T}\tau_{w}\left(t\right)\;dt$	Pa	Reflects the magnitude of the component of wall shear stress perpendicular to its average direction. High transWSS indicates a significant deviation from the average direction, usually associated with endothelial dysfunction, atherosclerotic plaque formation, and increased risk of aneurysm rupture
WSSG	$WSSG=\nabla_{\text{wall}}\left|\tau_{w}\right|$	Pa/m	Used to measure rapid changes in the local spatial distribution of wall shear stress, related to atherosclerosis-prone areas and aneurysm deformation
ECAP	$ECAP=\frac{OSI}{TAWSS}$	1/Pa	Used to assess the local blood flow environment’s potential to activate or damage endothelial cells, evaluating the likelihood of thrombus deposition
Q-criterion	$Q=\frac{1}{2}\left(\left|\Omega {|}^{2}\left.-\right|S{|}^{2}\right.\right)$ $\Omega =\frac{1}{2}\left(\nabla u-{\left(\nabla u\right)}^{T}\right),$ $S=\frac{1}{2}\left(\nabla u+{\left(\nabla u\right)}^{T}\right)$	s^-2^	Used to detect the location and intensity of vortex structures in the flow field, analyzing vortex activity and flow instability under complex flow conditions such as aneurysms or stenoses
Surface Area Ratio (SAR)	$SAR‐TAWSS=\frac{\text{Area}\left\{x\in \text{wall}:TAWSS\left(x\right)\leq \tau_{th}\right\}}{\text{Total}\;\text{Wall}\;\text{Area}}$ (Taking SAR-TAWSS as an example)	dimensionless	Reflects the proportion of the vessel surface areas with low TAWSS or high OSI relative to the total vessel surface area, aiding in the risk segmentation of arterial lesions or stent intervention areas
Vorticity	ω=∇×u	1/s	Indicates the degree of fluid rotation; high vorticity regions typically form at aneurysms and stenoses, suggesting mixed flow, backflow, and potential turbulence
Energy Loss	$\text{Energy Loss}=\sum_{\text{inlets}}\left(TP\times Q\right)-\sum_{\text{outlets}}\left(TP\times Q\right)$ $TP=p+\frac{1}{2}\rho \left|v\right.{|}^{2}$	W	Reflects energy loss due to viscosity and turbulence in flow, commonly associated with vessel stenosis or valvular dysfunction, leading to decreased efficiency

### 3.2 *In Vitro* experiment

Simulations based solely on CFD may easily lead to inaccurate results due to differences in vascular model processing methods or boundary condition settings by different researchers or clinicians. Consequently, verification with *in vitro* MCL systems becomes an essential auxiliary step, helping to confirm the fidelity of CFD results. Chen et al. (2022); Liang et al. (2022) study validated the accuracy of CFD numerical simulations using an *in vitro* modeling approach. The results showed that the average flow distribution ratio (FDR) difference between the CFD simulations and the standard data was 2.4% ± 1.70%. The comparison primarily employed paired t-tests to assess the statistical differences between the two methods (with a significance level set at *p* < 0.05), thereby confirming that, with proper selection of patient-specific geometries and physical properties (such as using a hyperelastic material model), the CFD simulation results regarding pressure waveforms and flow distribution are highly consistent with the *in vitro* experimental data.

The basic procedure of *in vitro* simulation experiments typically includes obtaining patient-specific vascular geometrical data through medical imaging (e.g., CT or MRI), followed by personalized 3D reconstruction using software such as Geomagic Wrap, and then manufacturing the vascular model via 3D printing. Silicone materials, such as Sylgard 184, are commonly used to construct vascular models, exhibiting an elastic modulus between two and 8 MPa, a Poisson’s ratio of approximately 0.49, and a density around 1,060 kg/m^3^ (Liang et al., 2022; Chen et al., 2022). Alternatively, transparent Plexiglass can be utilized for flow visualization, with an elastic modulus ranging from 2.5 to 3 GPa and a Poisson’s ratio of approximately 0.35 (Liang et al., 2022). Blood analog fluids typically comprise glycerin-water solutions (density: 1,050–1,060 kg/m^3^; viscosity: 3.5–4.1 mPa·s) to replicate the rheological properties of blood (Jamiolkowski et al., 2020; Espa et al., 2019).

**FIGURE 4:**
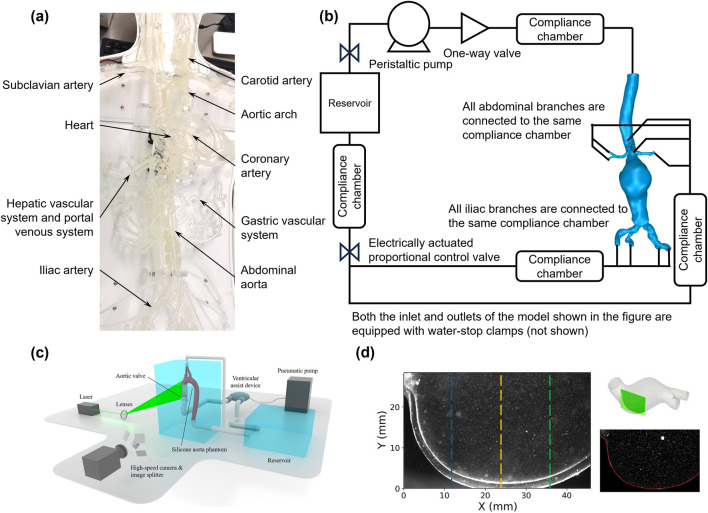
Experimental methods for hemodynamic analysis using physical models. **(a)** Setup of a physical in vitro vascular model; **(b)** Simulated circulation loop for an abdominal aortic aneurysm; **(c)** Schematic diagram of the 3D-PIV setup, consisting of a simulated circulation loop with electrical and optical components (Zeugin et al., 2024); **(d)** LED-PIV experimental results for two regions of interest in a patient’s actual AAA model (Bardi et al., 2024).

The distal vascular resistance and compliance of the vascular system are usually modeled using a three-element Windkessel model (Diamond and Forrester, 1972; Shaw et al., 2002). The compliance chamber utilizes the compressibility of air to simulate vascular compliance, whereas the glycerin-water solution flow simulates the viscous resistance of blood flow (Bardi et al., 2024; Salman et al., 2019; Kolli et al., 2016; Liang et al., 2023). Adjusting the pressure and volume within the air chamber precisely controls system compliance, thus simulating vascular elasticity characteristics under various physiological or pathological conditions (Shaw et al., 2002; Alfonso et al., 1994). After establishing the MCL, experimental data such as velocity fields can be measured using PIV or Doppler ultrasound, and real-time pressure waveforms, WSS, and flow rate data can be recorded using pressure sensors (Jamiolkowski et al., 2020; Espa et al., 2019; Rotman et al., 2019). The experimental data can be processed by filtering techniques (e.g., Savitzky-Golay filter) to remove high-frequency noise originating from the experimental setup and environment, ensuring accurate comparisons between experimental and CFD simulation results (He et al., 2024; Liang et al., 2022; Zhao et al., 2021; Jamiolkowski et al., 2020). This approach provides precise evidence for subsequent aneurysm diagnosis and treatment. However, it is noteworthy that many researchers currently restrict their focus to local vascular regions for *in vitro* validation or CFD simulations. Since the experimental scope significantly impacts simulation and numerical results, such as WSS and velocity distributions, attention should be paid to incorporating broader vascular pathways rather than being limited solely to lesion sites during numerical and experimental analyses. Figure 4 illustrates the general procedure and results processing of *in vitro* modeling.

Nevertheless, *in vitro* simulations still present several limitations. Existing materials struggle to fully replicate the nonlinear elasticity, anisotropy, and biological responses of real blood vessels. Furthermore, accurately simulating the mechanical characteristics of vascular intima, media, and adventitia, as well as plaque and thrombus, remains challenging *in vitro*. Therefore, investigating more realistic *in vitro* simulation materials is a crucial area for future exploration.

### 3.3 4D-flow MRI

4D-flow MRI has emerged as a non-invasive imaging modality for hemodynamic evaluation, providing time-resolved, three-dimensional velocity fields across the cardiac cycle through the application of velocity-encoding gradients integrated into phase-contrast MR imaging (Bissell et al., 2023). This technique allows for comprehensive quantification of hemodynamic parameters, including velocity, WSS, OSI, RRT, and pulse wave velocity (PWV), making it highly suitable for detailed assessment of complex flow patterns encountered in intracranial aneurysms (Peng et al., 2025; Schnell et al., 2012; Liu et al., 2018).

Recent studies have validated the accuracy and reliability of 4D-flow MRI-derived hemodynamic parameters through comparison with numerical simulation, *in vitro* experiment, and standard clinical imaging protocols. For example, Liu et al. (2018) developed an accelerated 4D-flow MRI approach combining advanced undersampling (CIRCUS) with compressed sensing reconstruction, achieving high temporal resolution (below 30 m) within clinically feasible scan times (∼5 min). Their work demonstrated robust qualitative and quantitative agreement with conventional imaging methods in both healthy volunteers and intracranial aneurysm patients, highlighting its ability to accurately visualize complex flow structures such as intra-aneurysmal vortices and recirculating flow patterns (Liu et al., 2018). Furthermore, Ferdian et al. (2022) introduced WSSNet, a deep-learning model specifically designed to improve WSS estimation accuracy from clinical-resolution 4D-flow MRI data, which significantly correlated with CFD-derived results (r = 0.92), addressing key challenges related to spatial resolution limitations.

Clinical applications of 4D-flow MRI have expanded significantly, particularly within aneurysm 00research. Misaki et al. (2021) validated the clinical utility of intracranial 4D-flow MRI by demonstrating strong correlations between MRI-derived flow parameters and CFD-simulated results, particularly in predicting rupture risk associated with abnormal hemodynamic features in aneurysms. Similarly, Koizumi et al. (2024) introduced the aneurysm damping index (ADI), an innovative parameter derived from 4D-flow MRI data, quantifying flow-induced mechanical energy attenuation within aneurysms, thereby offering new insights into aneurysmal wall compliance and stiffness, important markers for aneurysm stability. Brindise et al. (2019) further confirmed the robustness of dimensionless hemodynamic indices such as OSI across various modalities, underscoring the potential of 4D-flow MRI to reliably capture clinically relevant hemodynamic metrics even under spatial resolution constraints.

Looking forward, advancements in acquisition and reconstruction techniques, including multi-VENC encoding, compressed sensing, and machine-learning-based image reconstruction, will likely further enhance the capability of 4D-flow MRI to detect subtle yet clinically meaningful flow disturbances (Bissell et al., 2023; Brindise et al., 2019; Gao et al., 2019; Liu et al., 2018). These developments, coupled with ongoing standardization efforts detailed in recent expert consensus documents, suggest a promising trajectory toward widespread clinical adoption. Consequently, 4D-flow MRI is anticipated to play a pivotal role in future intracranial aneurysm management by improving patient-specific risk stratification, facilitating targeted therapeutic decisions, and enabling more precise longitudinal follow-up of aneurysm progression.

### 3.4 New techniques for predicting flow fields: deep learning

As mentioned in the previous section, obtaining more accurate simulation results requires developing more comprehensive mechanical models and precise boundary conditions. However, this approach significantly increases computational cost and time consumption, severely limiting clinical applications. Balancing computational efficiency and accuracy of results remains a critical issue for researchers.

**FIGURE 5:**
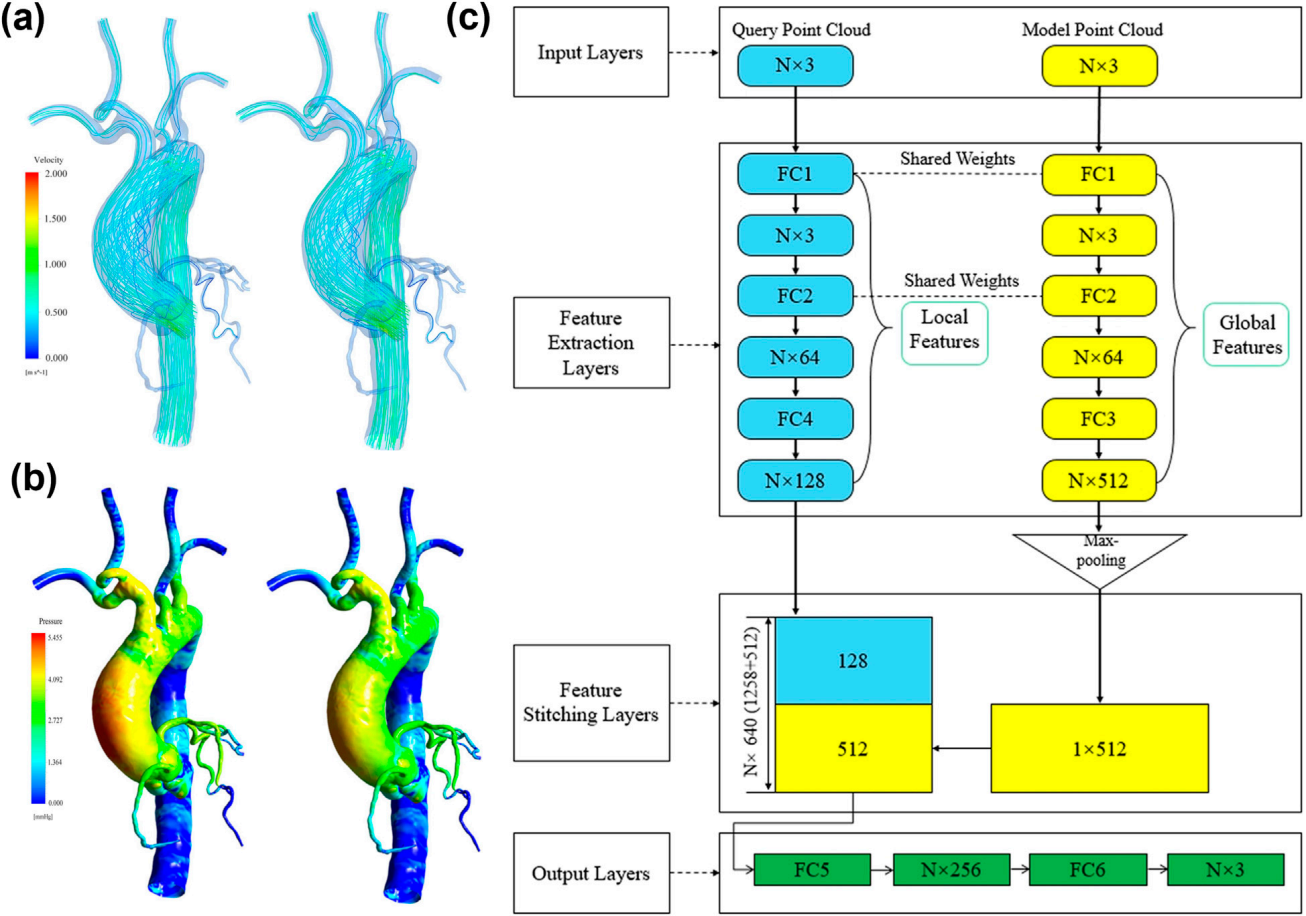
Deep learning-based reconstruction of coronary and aortic flow fields. **(a)** Velocity field reconstruction: CFD results on the left, deep learning results on the right; **(b)** Wall shear stress reconstruction: CFD results on the left, deep learning results on the right; **(c)** Deep learning network architecture for flow field reconstruction proposed by (Li et al., 2021).

In recent years, the integration of DL and CFD has become an essential trend in hemodynamic research. Although traditional CFD simulations hold irreplaceable precision in biomedical engineering, the incorporation of DL methods, particularly frameworks such as PINN, CNN, and GCN, has enabled researchers to predict and analyze hemodynamics quickly and accurately. Figure 5 summarizes current studies on aneurysmal hemodynamic characteristics based on deep learning methods, including flow field generation using deep learning, automatic segmentation and detection of aneurysm morphology from medical images, and risk prediction models for aneurysms. In coronary artery simulation, Alzhanov et al. (2024a), Alzhanov et al. (2024b) developed a hybrid CFD-PINN framework. By embedding the incompressible Navier-Stokes equations directly into the neural network training process, their method ensures mass and momentum conservation during training. This approach not only delivers accurate individualized predictions but also significantly reduces computational costs, representing a promising non-invasive functional diagnostic tool. Meanwhile, Suk et al. (2024a), Suk et al. (2024b) proposed a novel method based on SE (3)-equivariant Graph Convolutional Networks (GEM-GCN). By explicitly considering the local geometry and connectivity of mesh surfaces and using group-equivariant convolutions, this approach effectively captures detailed flow characteristics on vascular surfaces, enabling rapid and precise estimation of coronary artery WSS. This method demonstrates robustness to complex topologies and arbitrary spatial transformations, achieving approximately 7.6% error, indicating strong potential for clinical real-time assessment. Additionally, a comprehensive image-to-flow processing pipeline has received increased attention. Yao et al. (Yao et al., 2024) developed the Image2Flow network, ingeniously combining 3D CNN and GCN to directly segment pulmonary arteries from cardiac MRI images and estimate corresponding CFD flow fields rapidly and automatically. The significant advantage lies in its extremely fast processing (only hundreds of milliseconds) while maintaining good balance between segmentation accuracy (Dice ≈0.9) and flow prediction error (∼10%), promising widespread application in cardiopulmonary disease diagnosis and treatment processes.

Moreover, research efforts also focus on combining CFD simulations with structural analysis and machine learning to enhance clinical predictions of disease risk. Siogkas et al. (2024) integrated CFD-derived hemodynamic indices with structural simulation-derived local stresses to predict carotid plaque rupture risk using GBT, achieving diagnostic accuracy up to 88%. This model utilizes realistic pulsatile blood flow boundary conditions generated from 3D MRI data and ultrasound-measured flow waveforms, accurately simulating physiological environments, offering practical value for stroke risk prediction. Furthermore, considering the clinical importance of iFR, Liu J. et al. (2024), Liu X. et al. (2024) introduced a simplified model combining DL and physiological regulatory mechanisms to rapidly estimate non-invasive iFR. Trained on real clinical patient coronary CTA data and high-precision CFD simulations, their deep neural network predicts resistance due to coronary stenosis. Coupled with microcirculation auto-regulatory mechanisms, this method enables fast, non-invasive iFR (iFRCT) calculation with a diagnostic accuracy of up to 88.3%. Explicit consideration of physiological regulatory mechanisms enhances the physiological plausibility and clinical applicability of this model.

The studies mentioned above also highlight an increasing emphasis on interpretability. For example, the hybrid CNN-GCN model by Yao et al. (Yao et al., 2024) explicitly integrates spatial and vascular morphology information, providing a geometric perspective of interpretability, despite the absence of detailed feature attribution analysis. In contrast, Alamir et al. (2024) utilized integrated gradient methods to attribute neural network features, explicitly revealing the significance of vessel radius and inflow velocity in determining coronary artery WSS distributions. These approaches shift DL models from complete “black boxes” towards interpretability, supporting clinical decision-making more effectively. However, it is noteworthy that most researchers developing DL algorithms have not utilized high-fidelity simulations during dataset preparation, such as neglecting pulsatile flow, non-Newtonian blood properties, or realistic boundary conditions. For DL to achieve more precise blood flow simulations, high-fidelity simulation datasets or actual patient-measured data are necessary prerequisites. Additionally, many studies train models on local hemodynamic data while ignoring accuracy on large-scale models. Given physiological considerations, rapid flow field computation, lesion detection, and risk prediction for large-scale vessels (such as intracranial arteries, coronary arteries, or the entire aorta) deserve further attention.

To further mitigate the overfitting risk associated with small-sample training conditions and enhance model generalizability, current studies are increasingly incorporating hybrid strategies that combine data augmentation, transfer learning, and multi-task learning. For example, in the context of vascular flow simulation, Yao et al. (2024) employed self-supervised pretraining on synthetic flow data, followed by fine-tuning on patient-specific geometries, which significantly improved prediction accuracy with limited real-world samples. Additionally, methods like Bayesian Neural Networks (BNNs) and Monte Carlo Dropout (Gal and Ghahramani, 2015) have been adopted to quantify uncertainty in predictions and regularize model weights, thus preventing overfitting. From the interpretability perspective, techniques such as Integrated Gradients, SHAP (SHapley Additive exPlanations), and attention heatmaps are increasingly utilized to expose model decision processes, allowing clinicians to verify whether predictions align with known hemodynamic risk factors (e.g., low WSS, high OSI). Importantly, as suggested by Alamir et al. (2024), these interpretability tools not only identify which geometric or flow-related features contribute most to predictions but also support clinical trust-building by providing biologically meaningful rationales behind each diagnostic decision. Going forward, the integration of interpretable physics-constrained models, such as KANs and PINNs, with interactive visualization dashboards for clinical end-users, may offer an effective pathway toward regulatory acceptance and real-world adoption (Liu Z. et al., 2024; Liu J. et al., 2024; Ranasinghe et al., 2024).

In summary, recent studies applying DL to CFD-based hemodynamics exhibit three core trends: first, physics-informed or constrained DL (e.g., PINN) clearly shows advantages in accuracy and physical consistency; second, computational efficiency is significantly improved, greatly facilitating real-time or near-real-time applications and clinical feasibility; lastly, increased attention is being paid to model interpretability and decision-support capabilities, promoting model transparency and clinical acceptance. These innovations indicate that DL techniques are gradually moving hemodynamic simulations towards real-time, precise, and personalized clinical applications, potentially revolutionizing cardiovascular disease diagnostics and treatment paradigms in the future.

## 4 Conclusion

Significant advancements have been achieved in aneurysm hemodynamics research over the past few decades, uncovering the critical roles mechanical factors play in aneurysm initiation, progression, and rupture through multidisciplinary integration. This review systematically summarizes the heterogeneity observed across aneurysms located at various anatomical sites concerning morphological characteristics, mechanical environments, and mechanisms of thrombus formation. Particular emphasis has been placed on the complex associations between key hemodynamic parameters and aneurysm progression.

Integration of numerical simulations and *in vitro* experiments has provided essential tools for high-precision hemodynamic analyses, while the incorporation of DL techniques has substantially enhanced computational efficiency and clinical translation potential. Nonetheless, current research still encounters numerous challenges: rigid wall simplifications, uncertainties in boundary conditions within CFD simulations may introduce inaccuracies; DL models heavily rely on high-quality training datasets and suffer from insufficient interpretability; and biomimetic *in vitro* experimental materials often fail to replicate authentic vascular biomechanical properties. Furthermore, controversies surrounding the high/low WSS hypothesis and incomplete elucidation of mechanobiological coupling mechanisms necessitate integrated multi-scale and multi-omics studies.

Future research could benefit from further enhancing multidisciplinary collaboration, potentially integrating mechanobiology, multi-omics analyses, and artificial intelligence technologies to explore the dynamic mechanisms through which mechanical stimuli might influence aneurysm progression via endothelial cell signaling pathways. It may also be valuable to consider the development of patient-specific “digital twin” platforms that incorporate real-time imaging, blood flow simulations, and surgical planning, which could contribute to establishing a new paradigm for personalized aneurysm treatment.

## Glossary

AAA: Abdominal Aortic Aneurysm
ASI: Aneurysm Shape Index
BDF2: Backward Differentiation Formula 2
CAA: Coronary Artery Aneurysm
CE: Cross-sectional Eccentricity
CFD: Computational Fluid Dynamics
CNN: Convolutional Neural Networks
CTA: Computed Tomography Angiography
DL: Deep Learning
DNR: Dome-Neck Ratio
DSA: Digital Subtraction Angiography
ECAP: Endothelial Cell Activation Potential
FSI: Fluid-Structure Interaction
FVM: Finite Volume Method
GBT: Gradient Boosting Trees
GCN: Graph Convolutional Networks
IAs: Intracranial Aneurysm
iFR: Instantaneous wave-free Ratio
ZILT: Intraluminal Thrombus
LE: Longitudinal Eccentricity
LES: Large Eddy Simulation
MCL: Mock Circulation Loop
Micro-CT: Micro Computed Tomography
MMP: Matrix Metalloproteinase
MRA: Magnetic Resonance Angiography
MRI: Magnetic Resonance Imaging
NO: Nitric Oxide
OSI: Oscillatory Shear Index
PINN: Physics-Informed Neural Networks
PIV: Particle Image Velocimetry
RBC: Red Blood Cell
RRT: Relative Residence Time
SR: SizZe Ratio
TAA: Thoracic Aortic Aneurysm
TAWSS: Time-Averaged Wall Shear Stress
UI: Undulation Index
vWF: von Willebrand Factor
WSS: Wall Shear Stress
